# Comparative Clinicopathological Evaluation of the Ocular Surface in Newly Diagnosed Patients of Hyperthyroidism and Hypothyroidism Compared to Healthy Subjects

**DOI:** 10.7759/cureus.23890

**Published:** 2022-04-06

**Authors:** Nidhi Paharia, Indu Arora, Nikhil Agrawal, Abha Mathur, Shruti Agrawal

**Affiliations:** 1 Ophthalmology, Mahatma Gandhi Medical College and Research Institute, Jaipur, IND; 2 Ophthalmology, All India Institute of Medical Sciences, Jodhpur, Jodhpur, IND; 3 Pathology and Laboratory Medicine, Mahatma Gandhi Medical College and Research Institute, Jaipur, IND; 4 Pathology, All India Institute of Medical Sciences, Rishikesh, Rishikesh, IND

**Keywords:** squamous metaplasia, impression cytology, ocular surface disease, dry eyes, non-inflammatory thyroid eye disease

## Abstract

Introduction

Thyroid eye disease is a multifactorial disorder affecting the tear film and ocular surface, which leads to dry eye. The aim of the study is to evaluate the tear function test and ocular surface changes clinically and to correlate pathologically using impression cytology in recently diagnosed cases of thyroid dysfunction, including both hyperthyroid and hypothyroid patients, and to compare the results with healthy subjects.

Methods

This was a cross-sectional analytical study. Thirty diagnosed patients with hyperthyroidism and hypothyroidism, each within three months, and 30 healthy age-matched controls were included. All patients and controls underwent an assessment of proptosis, palpebral fissure height (PFH), tear function test, Ocular Surface Disease Index (OSDI) questionnaire, Schirmer’s test, tear break-up time (TBUT), and impression cytology for ocular surface assessment. These parameters were compared between cases and controls. Standard statistical analysis was used.

Results

Mean proptosis and PFH showed no significant difference among groups (P-value = .071). The mean value of OSDI was 42.33+22.67, 41.15+16.03, and 29.33+6.84 in the hyperthyroid, hypothyroid, and controls groups, respectively; the difference being statistically significant (p=0.001). Mean TBUT was 7.13+3.28 sec, 6.38+2.46 sec, and 11.15+2.39 sec in hyperthyroidism, hypothyroidism, and controls, respectively (p=0.001). The mean value of the Schirmer tear test was 12.93+5.81 mm, 13.30+4.44 mm, and 17.55+7.35 mm in hyperthyroidism, hypothyroidism, and controls, respectively (p=0.001). Eighty percent (80%) of patients in the hyperthyroidism group had grade 2-3 squamous metaplasia as compared to 70% in hypothyroidism patients and 24.4% in controls, signifying ocular surface damage (p<0.05).

Conclusions

The mean PFH and proptosis did not differ between the three groups. However, increased OSDI score, decreased Schirmer’s test value, decreased TBUT, and grades 2-3 squamous metaplasia in patients with thyroid dysfunction suggest the presence of dry eyes. Despite no clinically visible signs of thyroid ophthalmopathy, there is ocular surface damage right from the early stages of thyroid dysfunction, possibly attributable to the evaporative mechanism as well as ocular surface inflammation and instability of the tear film.

## Introduction

Thyroid disorders, both hyperthyroidism and hypothyroidism, are commonly prevalent worldwide, often affecting females more than males [1-2]. Thyroid disorders are often associated with ophthalmic manifestations, commonly referred to as thyroid eye disease (TED) or thyroid-associated ophthalmopathy (TAO). The prevalence of TED in hypothyroidism is 0.2-8.6%, whereas, in hyperthyroid patients, it is as high as 50% [3-5]. The approximate time from the onset of thyroid disease to the clinical manifestation of TED is 18 months [3-6]. Dry eyes are one of the most common manifestations in patients with TED, with a reported incidence varying between 23% and 96% [2-3].

TED is a multifactorial disease caused by increased surface evaporation of the tear film due to mechanical factors (lid retraction, increased palpebral fissure height, and exophthalmos), altered tear production due to immunological factors (ocular surface damage, alteration in tear film composition, and damage to the lacrimal glands [6-7]. According to the literature, 37% of patients with Hashimoto’s disease and 72% of patients with Grave’s disease have reported symptoms of dry eyes [8-9].

Conjunctival impression cytology, first introduced by Egbert et al. in 1977 [10], is a non-invasive, simple technique that provides information about the morphology of the superficial layers of the ocular surface [11-12]. It has been reported that ocular surface inflammation results in squamous metaplasia of the conjunctival epithelium, which can be demonstrated in conjunctival impression cytology. It may be the earliest clinical sign of Graves’ disease before the onset of clinically evident TAO [13-16]. Although the association of dry eyes with hyperthyroidism is well-documented [13-15], there is a paucity of literature documenting the presence of dry eyes in hypothyroid patients [9,16-17].

The study aimed to determine the presence of ocular surface inflammation in recently diagnosed patients with hypothyroidism and hyperthyroidism and to correlate the clinical findings pathologically with the help of impression cytology,

## Materials and methods

This cross-sectional study was conducted in the department of ophthalmology at a tertiary eye center in Western India following the tenets of the declaration of Helinski. Prior approval for the study was obtained from the Institutional Ethics Committee (MGMCH/IEC/JPR/2019/316) and informed consent was taken from the subjects before their enrolment. This study has been published on the Medrxiv preprint server in January 2020.

Patients who were diagnosed with hypothyroidism or hyperthyroidism within three months from the date of examination were selected randomly from the endocrinology department of the same hospital. Cases were defined as patients with primary hypo/hyperthyroidism diagnosed for the first time based on a combination of laboratory investigations and clinical diagnosis by an endocrinologist. Laboratory investigations included levels of T3, T4, and thyroid-stimulating hormone (TSH), thyroid peroxidase antibody (TPO), thyroid-stimulating hormone receptor antibodies, and thyroglobulin antibody.

The cases were further classified as group 1 (30 patients) - patients having hypothyroidism, and group 2 (30 patients) - those having hyperthyroidism. Patients with a history of intake of any form of topical ocular medications for pre-existing eye diseases, previous refractive error, prior ocular trauma or surgery, glaucoma, uveitis, contact lens wear, any known systemic autoimmune disorder, disorders affecting ocular motility (eg. myasthenia gravis, Lambert Eaton syndrome), diabetes mellitus, and hypertension were excluded from the study.

Age and gender-matched healthy controls (30 in number) were randomly chosen from among patients attending the ophthalmology clinic for refraction check without presenting symptoms of dry eyes and without any ocular comorbidity. This group was labeled as group 3. Only one eye of each patient was randomly selected for inclusion in the study in order to avoid selection bias.

Protocol for ocular evaluation

All the cases and controls underwent a complete ophthalmic examination of both eyes, including visual acuity testing, pupillary reaction, slit-lamp examination, applanation intraocular pressure measurement, fundus examination, and ocular motility assessment. Exophthalmometry was performed for both eyes of each patient for assessment of proptosis using Hertel’s exophthalmometer. Palpebral fissure height (PFH), the presence of lid retraction, and lid lag were documented for each eye. Lid retraction of the upper lid with a value of margin reflex distance of 3-5 mm was considered normal. Palpebral fissure height was evaluated by measuring the distance between the inferior margin of the eyelid to the superior eyelid margin. A value of 11-13 mm was considered normal.

All patients underwent the following tear function tests: ocular surface disease index (OSDI), Schirmer tear test, and tear film break-up time (TBUT). The symptoms of dry eye were assessed according to the OSDI questionnaire based on a one-week recall period. OSDI subscale scores can range from 0 to 100, with higher scores indicating more problems or symptoms. An OSDI score of 12 indicated a normal healthy eye, a score of 13-22 indicated a mild dry eye condition, 23-32 was regarded as a sign of a moderate dry eye condition, and a score of more than 33 was considered a sign of severe eye dryness. The index demonstrates sensitivity and specificity in distinguishing between normal subjects and patients with dry eye disease [13,18-20]. To measure TBUT, a fluorescein sodium strip moistened with a drop of non-preserved saline solution was applied to the inferior palpebral conjunctiva in each eye of the patients and the control subjects. The precorneal tear film was examined with a bio-microscope, and the elapsed time before the initial breakup, rupture of the tear film, or formations of tiny dry spots were recorded. A TBUT below 10 seconds was taken as abnormal. The Schirmer test was performed without anesthesia using Schirmer's strips. The strips were folded and placed over the temporal edge of the lower lid and wetting was measured after five minutes. A wetting of less than 10 mm was taken as abnormal on the Schirmer I tear test.

Methodology for conjunctival impression cytology

Conjunctival impression cytology was performed in all patients to study the morphologic characteristics of the ocular surface. Samples were collected from the temporal interpalpebral bulbar conjunctiva. A cellulose acetate filter (Millipore GS0.22μm, Bedford, Massachusets) was used to obtain samples. The filter paper was cut asymmetrically to form a quadrilateral with 4 mm in height and 5 and 6 mm in length for the temporal bulbar conjunctiva. The filter paper was placed keeping the matt surface on the conjunctival side, 3 mm behind the limbus, and gentle pressure was applied to provide better adherence to the conjunctival tissue. The paper was removed with non-toothed forceps, fixated in a solution of 1:1:20 glacial acetic acid, 37% formaldehyde, and 70% ethyl alcohol, and stained with periodic acid Schiff (PAS) and hematoxylin according to the protocol described by Tseng (1985) [11]. After mounting the paper, it was examined under the light microscope, and the cytologic changes were graded according to Nelson’s grading system (Table 1) [12].

**Table 1:** Nelson's grading system for conjunctival impression cytology PAS: periodic acid Schiff

Grade 0	Small and round epithelial cells with a nucleo-cytoplasmic ratio of 1:2 and eosinophilic cytoplasm; abundant, plump, oval goblet cells with intensely PAS-positive cytoplasm.
Grade 1	Slightly larger and more polygonal epithelial cells with a nucleo-cytoplasmic ratio of 1:3 and eosinophilic cytoplasm. There is a decrease in goblet cell numbers.
Grade 2	Larger and polygonal, occasionally multinucleated epithelial cells with variable staining cytoplasm and a nucleo-cytoplasmic ratio of 1:4-1:5. Smaller and less intensely PAS-positive goblet cells with poorly defined cellular borders markedly decreased in number.
Grade 3	Large polygonal epithelial cells with basophilic cytoplasm. Small, pyknotic, and, in many cells, completely absent nuclei with a nucleo-cytoplasmic ratio greater than 1:6. Goblet cells are absent.

Dry eyes were diagnosed on the basis of altered tear films indices and ocular surface alteration in patients with a thyroid disorder. Therefore, the main outcome measures in our study were to compare the tear film indices (OSDI scores, Schirmer test, TBUT) and impression cytology grading between cases (Groups I and II) and controls (Group III).

Statistical analysis

The data including demographic profile, tear film indices, and conjunctival impression cytology grading was entered in Microsoft Excel® (Microsoft Corporation, Redmond, WA). Statistical analysis was performed using the SPSS software (IBM Corp., Armonk, NY) version 19.0. The Kruskal-Wallis test was used to compare differences in the median of OSDI, TBUT, and the Schirmer tear test between groups. The impression cytology findings were compared using the chi-square test. Differences were considered statistically significant at a p-value of < 0.005.

## Results

The mean age of patients in Group I was 46.3 + 7.5 years, Group II was 47.3 + 8.5 years, and Group III was 44.6 + 6.4 years (Mean+SD). There was an obvious female predominance in the patient distribution in the cases as well as the controls with a male to female ratio of 1:3, 1:2, and 1:2 in groups I, II, and III, respectively (p=0.386). The dry eye symptoms were significantly higher in cases, i.e., dysthyroid patients as compared to the healthy controls. Findings of the ocular examination are presented in Table 2.

**Table 2:** Comparison of the ocular surface and dry eye disease assessment between the groups *IQR = Interquartile Range; #P value of < 0.05 considered significant; Kruskal Wallis test used

	Group 1 (Hypothyroidism, n=30) MEDIAN (IQR*)	Group 2 (Hyperthyroidism, n=30) Median(IQR)	Group 3 (Control,n=30) Median (IQR)	P-value#
Palpebral Fissure Height (mm)	12.20(1.0)	12.5(1.0)	12.00(1.0)	0.185
Proptosis	20.00(3.0)	20.0(2.0)	19.50(2.0)	0.479
Ocular Surface Disease Index (OSDI)	41.70(23.5)	44.90(32.5)	18.95(10.2)	<0.001
Tear Film Break-up Time (TBUT, Seconds)	6.00(3.3)	7.00(7.3)	12.00(2.0)	<0.001
Schirmer’s Test (Wetting of strip,mm)	13.00(4.3)	11.00(5.5)	15.50(10.8)	0.008

Both, Groups I and II had a higher mean palpebral fissure height as compared to Group III although the difference was not statistically significant. Similarly, exophthalmometry did not reveal statistically significant differences between the three groups (p = 0.479). Ocular surface and dry eye assessment findings are shown in Table 2. Groups I and II had a significantly greater number of patients with dry eye disease as compared to healthy controls, with the OSDI being higher and the TBUT and Shirmer’s test being significantly lower in the dysthyroid patients as compared to the controls (p<0.05).

**Figure 1:**
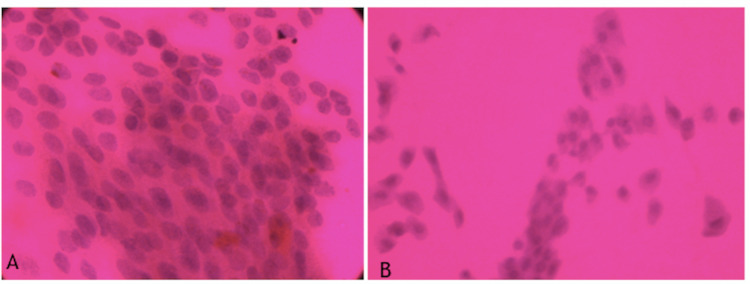
a. Grade 0: epithelial cells - small & round, nucleus: cytoplasmic (N/C) ratio 1:2 goblet cells: abundant, plump, oval with PAS-positive cytoplasm b. Grade 1: epithelial cells - slightly larger and more polygonal with an N/C ratio of 1:3. Goblet cell: decrease in cell number PAS: periodic acid Schiff

**Figure 2:**
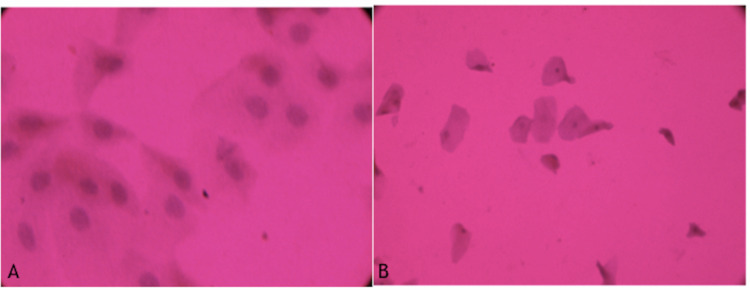
Grade 2: epithelial cell: larger and polygonal, with N/C ratio 1:4-1:5 with poorly defined cellular border

As shown in Table 3, the assessment of the conjunctival impression cytology specimen collected from temporal inter-palpebral conjunctiva showed 70% and 80% Grade II-III squamous metaplasia in hypothyroid and hyperthyroid patients, respectively, as compared to 26.6% in healthy controls (p=0.001) (Figure1, Figure 2).

**Table 3:** Incidence of squamous metaplasia on conjunctival impression cytology: comparison between the groups

	Group 1 (n=30) (Hypo)	Group 2 (n=30) (Hyper)	Group 3 (n=30) (Control)	P-value
Grade 0-1	9(30%)	6(20%)	22(73.3%)	<0.001
Grade 2-3	21(70%)	24(80%)	8(26.6%)	<0.001

## Discussion

In thyroid ophthalmopathy, proptosis and increased PFH have been conventionally considered to be the major contributing factor in the etiology of dry eyes by causing increased evaporation of the tear film due to impaired blinking [18]. Though mechanical factors contribute to the evaporative component of TED-associated dry eye, several other underlying factors have been studied and are known to aggravate diabetic eye disease (DED) [18-19]. Furthermore, it has been postulated that the degree of tear film evaporation is proportional to the ocular surface area exposed [19]. However, in the present study, a majority of the patients with both hyperthyroidism and hypothyroidism showed deranged tear function tests and the presence of squamous metaplasia on impression cytology even without the presence of proptosis and increased PFH. This suggests the role of an alternative mechanism in the pathogenesis of dry eyes in cases of thyroid dysfunction.

Ophthalmic evaluation of subjects showed that OSDI was in the severely impaired range in both hypothyroid and hyperthyroid patients, which was in concordance with the findings of Gurdal et al. [13,20] and Turkyilmaz et al. [21]. In asymptomatic patients with thyroid dysfunction, despite normal PFH, the OSDI score was high in comparison to healthy controls, correlating the presence of mild to moderate dry eye.

Schirmer’s score was also found to be reduced in patients with thyroid dysfunction as compared to healthy subjects in this study. This finding was congruous with the results reported by Eckstein et al. in their study [15]. They identified the presence of TSH receptors not only on the lacrimal gland but also in corneal and conjunctival epithelium, which acted as targets for autoantibodies in thyroid disease, resulting in lacrimal gland impairment and subsequent attenuation in tear secretion. Villani et al. concluded that a reduced Schirmer’s score in patients with Grave’s ophthalmopathy with normal PFH was due to diminished corneal sensitivity due to ocular surface inflammation explaining the occult character of dry eyes in these patients [22]. 

The tear breakup time (TBUT) was significantly decreased in hyperthyroid and hypothyroid patients in the present study, suggesting an unstable tear film in comparison to the control subjects. According to the U.S. National Eye Institute (NEI) Committee on dry eyes, the instability of tear film could be caused by hyperosmolarity of tear film resulting from a decrease in tear secretion due to a lacrimal gland disease or from an increase in tear film evaporation [18]. On the other hand, Kan et al. discovered the role of inflammation, in addition, to tear film hyperosmolarity in the development of dry eyes in TAO [16].

Impression cytology is the standard technique to study the effect of chronic inflammation in the form of squamous metaplasia and loss of goblet cells in ocular surface disorder. In the current study, a greater proportion of the patients with hyperthyroidism as well as hypothyroidism had Grade II-III squamous metaplasia in comparison to the healthy controls. Persistent hyperosmolarity of the tear film and ocular surface inflammation may cause pathological changes in corneal epithelium, such as increased desquamation, blunting and loss of microplicae, disruptions of the cell membrane, and cellular swelling with decreased goblet cell density. These cascades of events lead to squamous metaplasia, reduced goblet cell density, and larger polygonal epithelial cells with a decreased nucleo-cytoplasmic ratio that can be picked up by a meticulously performed impression cytology technique [23]. Besides, the presence of lymphocytes in conjunctival impression cytology as observed in patients with thyroid dysfunction as compared to controls further strengthen the role of ocular surface inflammation [20].

Hoffman et al. demonstrated that deficiency of thyroid hormone (TH) in hypothyroid patients predisposes to ocular surface structural changes [24]. The possible pathophysiology could be the increase in oxidative stress caused due to an alteration in oxidative metabolism by reduced levels of TH, which normally regulates the function of lacrimal glands, cornea, and conjunctiva through TSH receptors. Hence, a chronic low thyroxine level has an impact on both tear secretion and ocular surface inflammation with resultant alternation in TBUT and Schirmer’s test as confirmed by impression cytology findings [25-27].

**Figure 3:**
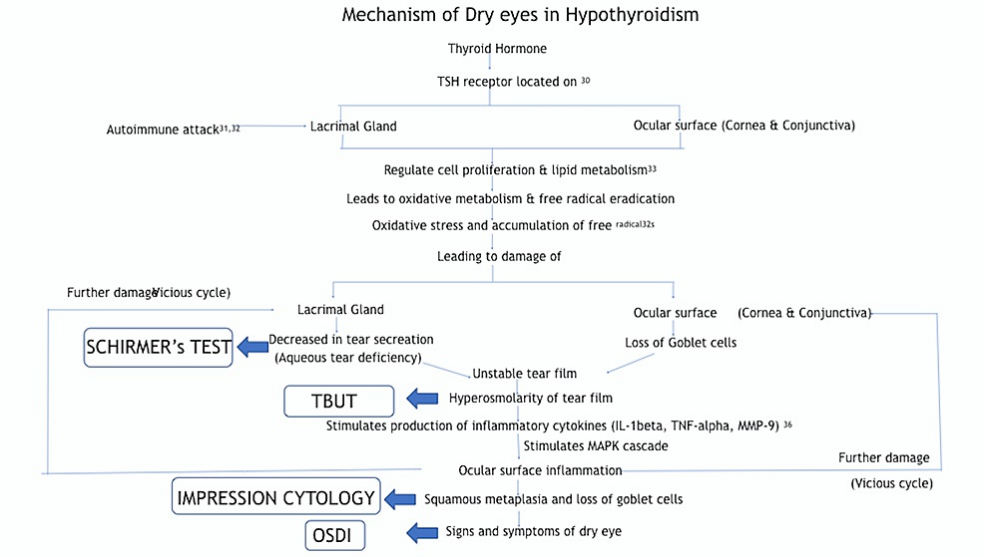
Proposed mechanism of dry eyes in hypothyroidism Proposed hypothesis by the authors

**Figure 4:**
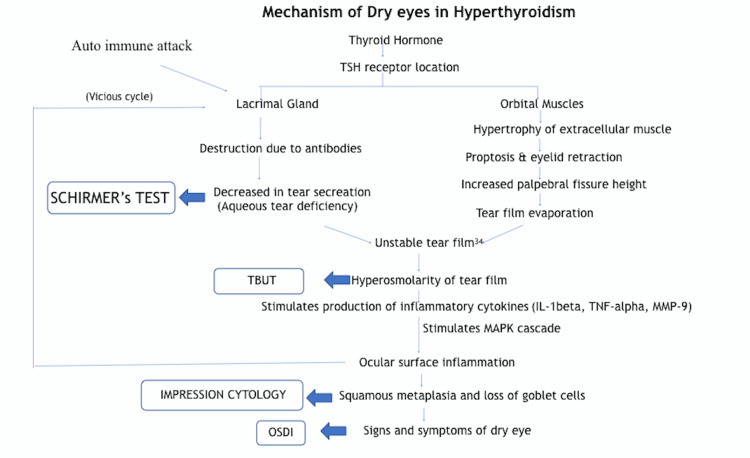
Proposed mechanism of dry eyes in hyperthyroidism Proposed hypothesis by the authors

The etiopathogenesis of dry eye related to TED has been investigated in several recent studies and has been summarised in Figure 3 and Figure 4. According to Luo et al., tear film hyperosmolarity has been shown to induce dry eye syndrome through inflammatory cytokines such as interleukin-1 (IL-1), tumor necrosis factor-alpha (TNF-alpha), and matrix metallopeptidase 9 (MMP-9), which activate the mitogen active protein kinase signal (MAPK) pathway leading to ocular surface damage and dry eyes [28]. Iskeleli et al. postulated that disruptions of the normal composition, quantity, and physiology of the ocular tear film can cause a vicious cycle of increased tear film evaporation and subsequent dry eye symptoms [29]. The present study, however, emphasizes the role of ocular surface inflammation as a causative factor for dry eyes because of the presence of an unstable tear film even in the absence of proptosis and increased PFH in patients with a thyroid disorder [17].

In view of our findings, we recommend that the thyroid profile should be performed in patients diagnosed with dry eye, especially among women of 30-50 years. Proactive ocular surface and dry eye evaluation should be performed in patients diagnosed with hyperthyroidism and hypothyroidism. In a high-volume OPD, TBUT should be performed in all patients with thyroid dysfunction. To summarize, an ophthalmologic referral is mandatory for a patient diagnosed with thyroid dysfunction.

The limitations of the study include a small sample size apart from lack of objective evaluation of meibomian glands, analysis of parameters of autoimmunity not done, non-availability of inflammatory markers in impression cytology, and non-measurement of hyperosmolarity of the tear film.

## Conclusions

Significant conjunctival squamous metaplasia is present in dysthyroid patients in contrast to euthyroid patients in spite of no significant difference in the mean PFH and proptosis between the two groups. We suggest that the deranged tear function in patients with thyroid dysfunction is attributed to ocular surface inflammation due to unstable tear film (hyperosmolarity) rather than tear film evaporation. Therefore, patients with thyroid dysfunction should be evaluated for the ocular surface disorder, as a timely intervention may prevent pathological changes.
